# On Medical Reform

**Published:** 1813-09

**Authors:** 


*To the Editors of the Medical and Physical Journal.*

GENTLEMEN,

HAVING perused the many excellent observations and opinions contained in the different Numbers of your enlightened Journal, on the subject of Medical Reform, I am rather surprised to find no notice taken of what I have great reason to believe is become almost a crying evil, which is destructive of many a patient, and highly injurious to the credit and character of many a physician. To guard against which, I hope the legislature will, in its wisdom, inflict a very heavy penalty on any one who shall in future venture to substitute, without leave of the prescriber, one medicine for another, in any prescription he shall undertake to prepare; or who shall not prepare it exactly according to the formula prescribed. For, whether from indolence, ignorance, or self-conceit it occurs, it is equally productive of the mischief above-mentioned; and from which I am sorry to have occasion to say, I, and many of my patients, have been great sufferers.

MEDICUS.

